# Effect of Ag Templates on the Formation of Au-Ag Hollow/Core-Shell Nanostructures

**DOI:** 10.1186/s11671-015-1141-7

**Published:** 2015-11-13

**Authors:** Chi-Hang Tsai, Shih-Yun Chen, Jenn-Ming Song, Mitsutaka Haruta, Hiroki Kurata

**Affiliations:** Graduate Institute of Applied Science and Technology, National Taiwan University of Science and Technology, Taipei, 106 Taiwan; Department of Materials Science and Engineering, National Taiwan University of Science and Technology, Taipei, 106 Taiwan; Department of Materials Science and Engineering, National Chung Hsing University, Taichung, 402 Taiwan; Institute for Chemical Research, Kyoto University, Kyoto, 611-0011 Japan

**Keywords:** Galvanic replacement, AuAg alloy rings, Ag@AuAg core-shell wires

## Abstract

Au-Ag alloy nanostructures with various shapes were synthesized using a successive reduction method in this study. By means of galvanic replacement, twined Ag nanoparticles (NPs) and single-crystalline Ag nanowires (NWs) were adopted as templates, respectively, and alloyed with the same amount of Au^+^ ions. High angle annular dark field-scanning TEM (HAADF-STEM) images observed from different rotation angles confirm that Ag NPs turned into AuAg alloy rings with an Au/Ag ratio of 1. The shifts of surface plasmon resonance and chemical composition reveal the evolution of the alloy ring formation. On the other hand, single-crystalline Ag NWs became Ag@AuAg core-shell wires instead of hollow nanostructure through a process of galvanic replacement. It is proposed that in addition to the ratio of Ag templates and Au ion additives, the twin boundaries of the Ag templates were the dominating factor causing hollow alloy nanostructures.

## Background

AuAg alloy nanostructures with zero or one dimension (0-D or 1-D) exhibit unique optical and catalytic properties and have diverse applications, e.g., catalytic converters and electrode catalysts for Li batteries [1–8]. Alloying of Au and Ag gives rise to the combination of performance between the metal components and has a variety of applications, including tunable localized surface plasmon resonance features [9], surface enhanced Raman scattering [10–12], optical imaging [13–15], photothermal therapy [16], and encapsulation [17]. As for the catalytic properties, previous reports show that AuAg alloy nanoparticles (NPs) exhibit superior catalytic activity for CO oxidation compared with pure Au and Ag NPs, also due to the synergistic effect [7, 8, 18].

The synthesis and the formation of alloy nanostructures have attracted considerable attention. The most common way to prepare AuAg nanostructures is through the chemical reduction method [19, 20]. AuAg NPs can be reduced from the solution with a mixture of Au and Ag ions containing precursors. In these cases, Ag NPs are usually used as the templates, and Au ions are added for alloying [19]. To synthesize AuAg alloy NPs, another method proposed by Crespo et al. is the mild decomposition of the single-source organometallic precursor [21]. In this case, the composition, size, as well as the surface plasmon resonance of AuAg NPs, can be easily controlled by adding different amounts of hexadecylamine (HDA). Crespo et al. [22] also suggested an organometallic approach by using HDA-capped Ag as templates. By controlling the concentration of [Au(C6F5)(tht)] (tht = tetrahydrothiophene), Ag@Au nanostructures with different shapes can be prepared, including circles, hexagons, triangles, truncated triangles [22].

Table 1 lists some relevant studies on the synthesis of AuAg nanostructures. These nanostructures can be categorized into two groups, viz., solid structure and hollow structure. It has been suggested that by adjusting the ratio of Ag templates and Au ion additives, Ag@Au or Ag@AuAg core-shell nanostructures can be obtained [19, 22, 23]. The concentration of the Au precursors plays an important role in the replacement reaction of Ag by Au, and the structure, morphology, as well as the composition, can thus be controlled. The excessive galvanic reaction between Au and Ag might give rise to the formation of hollow structures [6, 24–30], e.g., AuAg hollow particles and tubes. As also tabulated in Table 1, the characteristics of Ag templates, e.g., the existence of planar defects (twins in this case), size, and specific surface area of templates, may also affect the morphology of the AuAg nanostructures. However, there is still a lack of studies regarding Ag templates.

**Table 1:** A summary of prior works of Au-Ag alloy nanostructures

Structures		Ag template crystal	Ag template size	Ag specific surface area (nm^−1^)	AuAg nanostructure size (nm)	Ref.
	AuAg NPs	n/a	6.1 nm	0.98	13	19
	AuAg NPs	n/a	7.4 nm	0.81	16	19
	Ag@Au NPs	n/a	9.6 nm	0.63	15	19
Solid	AuAg NPs	n/a	20.7 nm	0.29	~20	20
	Ag@Au NPs	n/a	9.8 nm	0.61	Ag_core_ = 8.1, Au_shell_ = 6	22
	Ag@AuAg NPs	Single crystalline	18.6 nm	0.32	n/a	23
	Ag@AuAg NWs	Single crystalline	d = 90 nm h = 10 μm	0.02	Ag_core_ = 85, AuAg_shell_ = 6	this study
	AuAg hollow NPs	Twined	11 nm, 14 nm	0.42, 0.54	d_inner_ = 12.4, d_outer_ = 16.9	[24]
	AuAg hollow NPs	n/a	41 nm	0.14	46–53	[6]
	AuAg hollow NPs	Twined	53 nm	0.11	68	[25]
Hollow	AuAg hollow NPs	n/a	20–100 nm	0.3-0.06	d_inner_ = 88, d_outer_ = 128	[26]
	AuAg hollow NPs	Twined	75 nm	0.08	n/a	[27]
	AuAg nanotubes	Twined	d = 50 nm	n/a	n/a	[28]
	AuAg nanotubes	Twined	d = 30–60 nm h = 1-50 μm	0.06–0.13	n/a	[29, 30]
	AuAg nanorings	Twined	14 nm	0.42	d_inner_ = 2–4.5, d_outer_ = 12–15	this study

n/a means not available

In order to clarify the dominant factors determining the formation of hollow AuAg alloy nanostructures, two kinds of templates, with a variety of differences, twinned Ag NPs and single-crystalline Ag nanowires (NWs), were used. The same moles of Au ions were added for alloying. In combination with the cases given in Table 1, the effect of the Ag templates will be investigated. In addition, to study the hollow structure in depth, the HAADF images of the nanostructures from different angles will be observed.

## Methods

### Preparation of Au-Ag Nanostructures

**Fig. 1:**
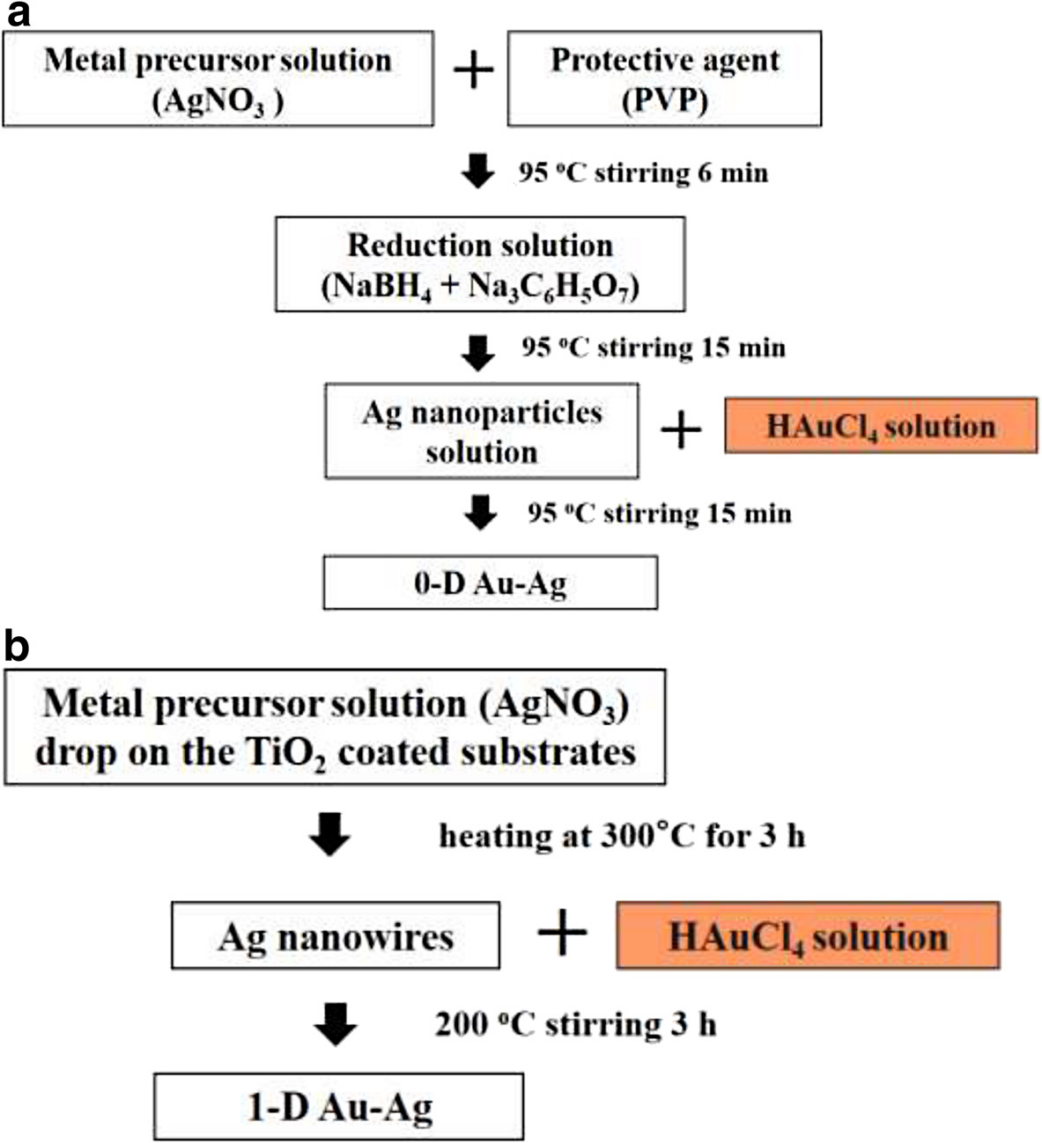
Synthetic process of **a** Au-Ag zero-dimensional structure and **b** Au-Ag one-dimensional structure

**Fig. 2:**
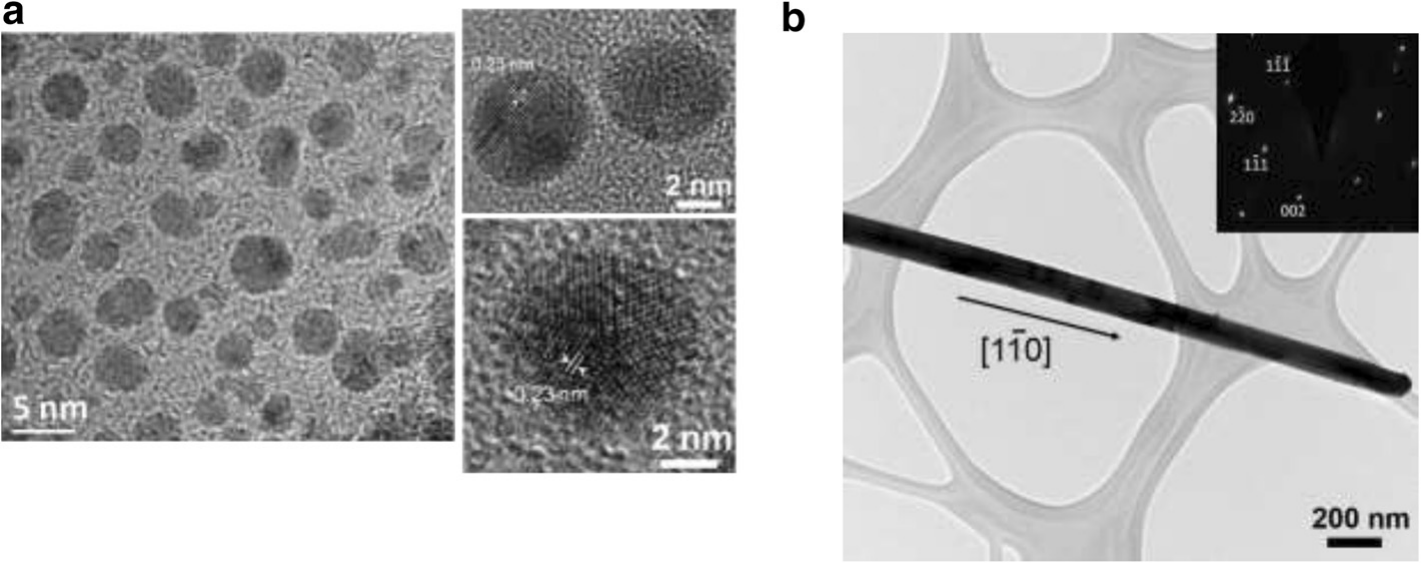
TEM images of templates **a** Ag NPs of zero-dimensional structure: (right) twined Ag NPs and (left) magnified images showing lattice fringes and **b** Ag NWs of one-dimensional structure and the inserted diffraction pattern

The processes for preparing Au-Ag nanostructures with different dimensions are illustrated in Fig. 1. AgNO_3_ and HAuCl_4_ aqueous solutions with the same concentrations (0.4 mM) are the precursors. Figure 1a shows the procedures for the synthesis of 0-D Au-Ag structures. The templates, Ag NPs, were obtained from the reduction of AgNO_3_ using a mixed solution with 1 wt% of NaBH_4_ and 1 wt% of Na_3_C_6_H_5_O_7_. 0.29 mM (MW 55,000 g/mol) PVP, the surfactant to prevent particle aggregation, was dissolved in water by stirring for 6 min at 95 °C. As indicated in Fig. 2a, b, the Ag NPs thus produced are heavily twinned with an average particle size of 14 nm. The interplanar distance of 0.23 nm can be referred to {111} planes of Ag. HAuCl_4_ aqueous solution was then mixed with the Ag NP suspensions and agitated at 95 °C for 15 min.

With respect to 1-D nanostructures, single-crystalline Ag NWs were prepared via thermally-assisted photoreduction; the procedures of which have been described in previous works [31]. Fifteen-microliter droplets of 0.1 M AgNO_3_ aqueous solution were dropped on the annealed TiO_2_ substrates, followed by a post-heat treatment in an IR furnace. The samples were heated isothermally at 300 °C for 3 h and then furnace-cooled. The diffraction pattern inserted in Fig. 2c demonstrates that the Ag NWs thus synthesized are single crystalline with the $\left[1\overline{1}0\right]$ growth direction. The average wire length and diameter are 10 μm and 90 nm, respectively. In order to form 1-D AuAg nanostructure, HAuCl_4_ aqueous solution was mixed with the Ag NW suspensions and agitated at 200 °C for 3 hr.

### Microstructural and Characteristic Analyses

The UV-visible spectra of the nanostructures solutions were measured by a spectrophotometer (Varian Cary 100 UV-Visible spectrometer) with a 10 mm quartz cell. The electron microscope used in this study was a TEM/STEM (TEM, JEM-2200FS, JEOL, Japan) operated at 200 kV, equipped with an energy dispersive X-ray spectroscopy (EDS). High angle annular dark field-scanning TEM (HAADF-STEM) images were collected over scattering angles from −30 to 30 mrad (*X* and *Y* axes).

## Results and Discussion

**Fig. 3:**
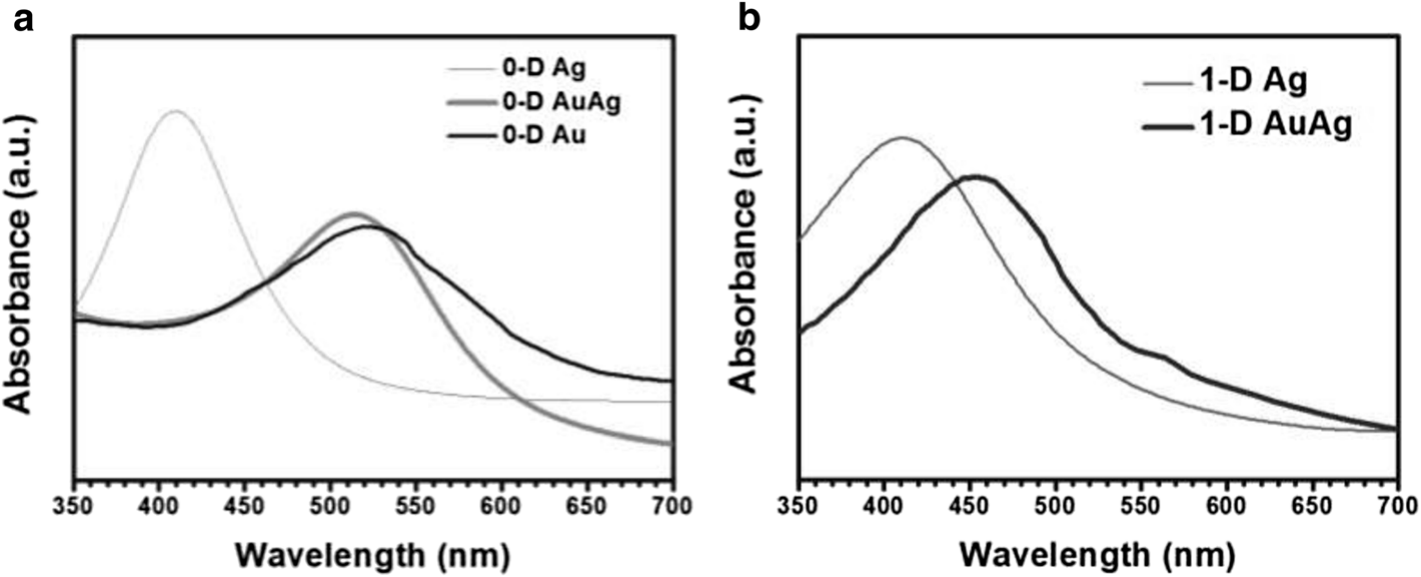
UV-vis absorption spectra: **a** zero-dimensional structure and **b** one-dimensional structure

Figure 3 illustrates the UV-visible absorption spectra of the templates and the Au-Ag nanostructured thus obtained. The absorption peaks centered at 407 nm for Ag NPs and 408 nm for Ag NWs, respectively, due to surface plasmon resonance, are identical to the reported value [25, 32–33]. After being reacted with HAuCl_4_ solution, a small red shift for the absorption peaks could be observed. As illustrated, the absorption peak was 506 nm for 0-D nanostructures, while that for 1-D nanostructures was 455 nm, and both the wavelengths were lower than Au NPs in the literature [34, 35] . The fact that the optical absorption spectrum shows only one plasmon band reveals that the surface of the 0-D and 1-D Au-Ag nanostructures was alloy rather than individual Au and Ag.

**Fig. 4:**
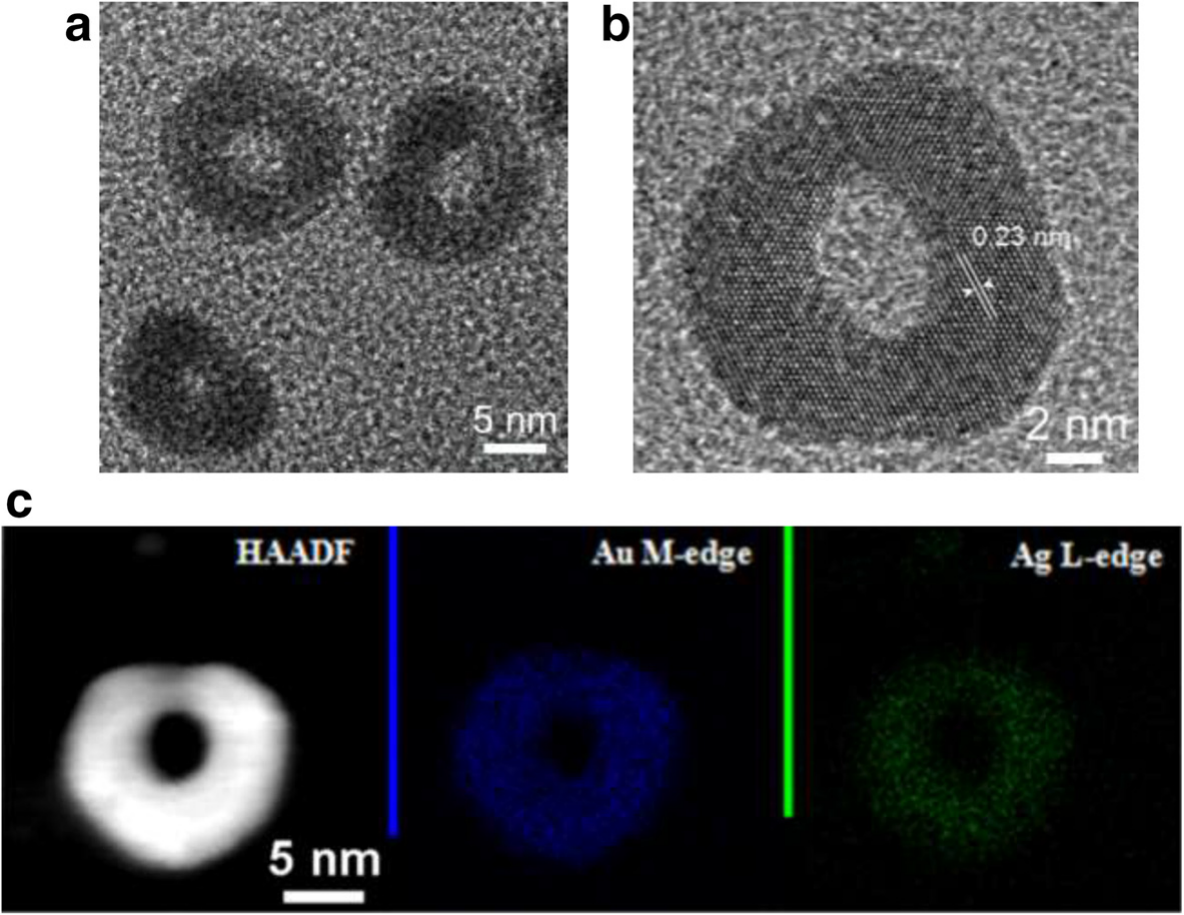
Microstructural observation results of the 0-D Au-Ag nanostructures: **a** TEM image and **b** HRTEM image and **c** HAADF image and EDS elemental mapping

Electron microscopic observation (Fig. 4) indicates that the 0-D nanostructures are hollow and exhibit an average size of 20 ± 3.5 nm, as well as an identical interplanar distance with Ag NPs (Fig. 4b). The HAADF image and EDS elemental mapping further verify that the nanostructures are composed of Au and Ag in the form of uniform alloy (Fig. 4c). EDS quantitative results show the Ag content of the hollow structures was about 51 at. % (Table 2). A recent report studied hollow AuAg alloy rings and the composition effect on the segregation of Au and Ag based on the EDS elemental mapping data [36]. However, our observation found no such segregation.

**Table 2:** EDS results of AuAg nanorings and the shell of Ag@AuAg NWs (at. %)

Element (at. %)	0-D AuAg	Shell of 1-D AuAg
Ag	51.03	56.18
Au	48.97	43.82

**Fig. 5:**
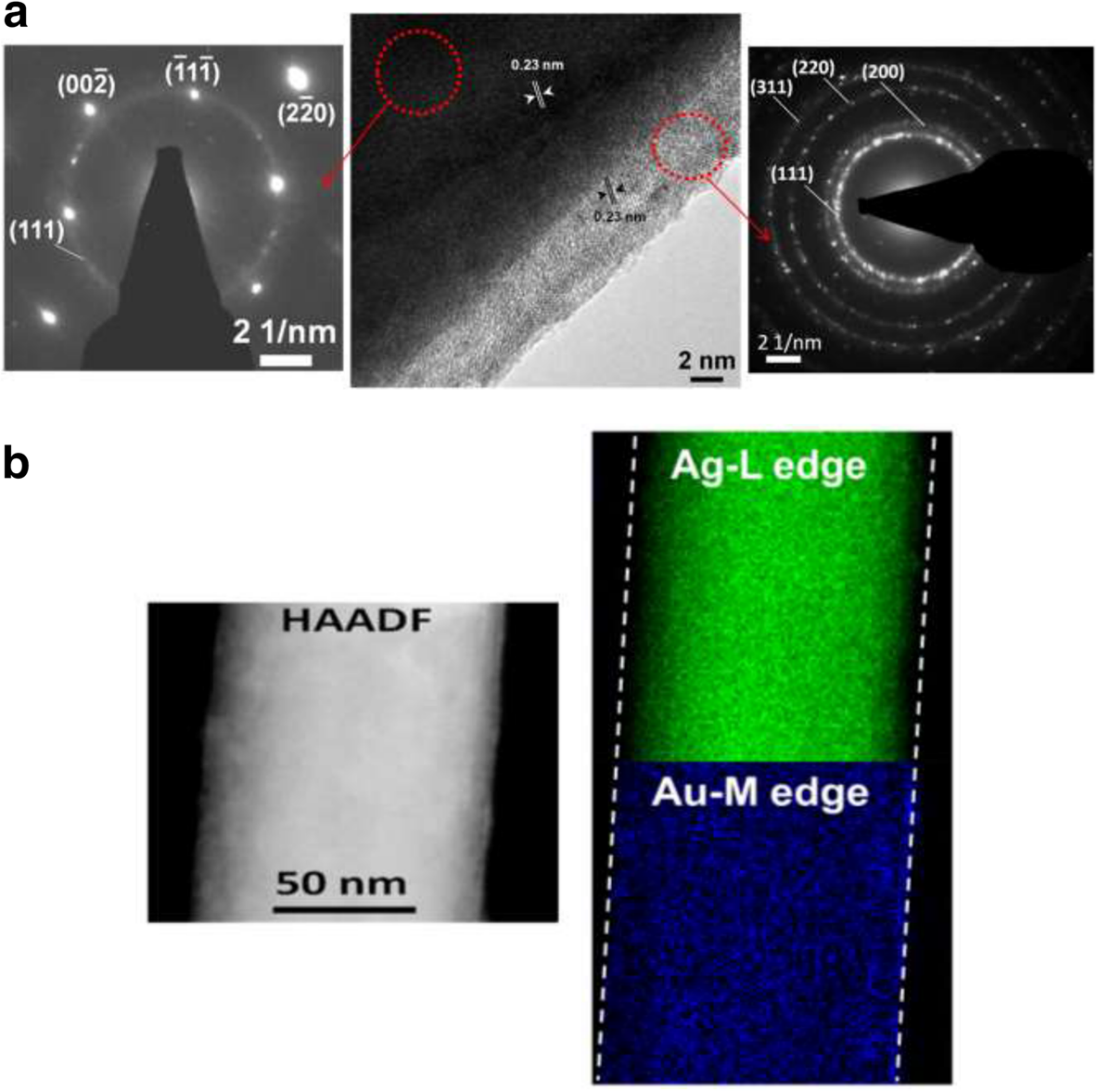
Microstructural observation results of the 1-D Au-Ag nanostructures: **a** HRTEM images and selected area diffraction patterns (SADP) and **b** HAADF image and EDS elemental mapping of Ag (upper) and Au (lower)

Moreover, high-resolution TEM (HRTEM) images taken from the near-edge regions of the 1-D Au-Ag nanostructures and corresponding selected area diffraction patterns (SADPs) given in Fig. 5a reveal that the core-shell feature of the 1-D nanostructures, of which the core was single-crystalline Ag wires, and the shell deposits, compact and attached firmly to the Ag surface, with thickness of 4 ± 0.3 nm comprised nanocrystals showing an interplanar distance of 0.23 nm. Similar to the hollow structure in Fig. 4, the HAADF image of the 1-D nanostructure as well as EDS elemental mapping further suggest the nanostructures are alloy of Au and Ag possessing Ag content of 56.2 at. % (Table 2). That the Ag signal at the edge region is less bright than Au further confirms the core-shell structural feature of the galvanic-reacted NWs. The above results may reveal that in the absence of surfactants and grain boundaries, a clean surface of single-crystalline Ag NWs is conductive to the precipitation of AuAg alloys.

**Fig. 6:**
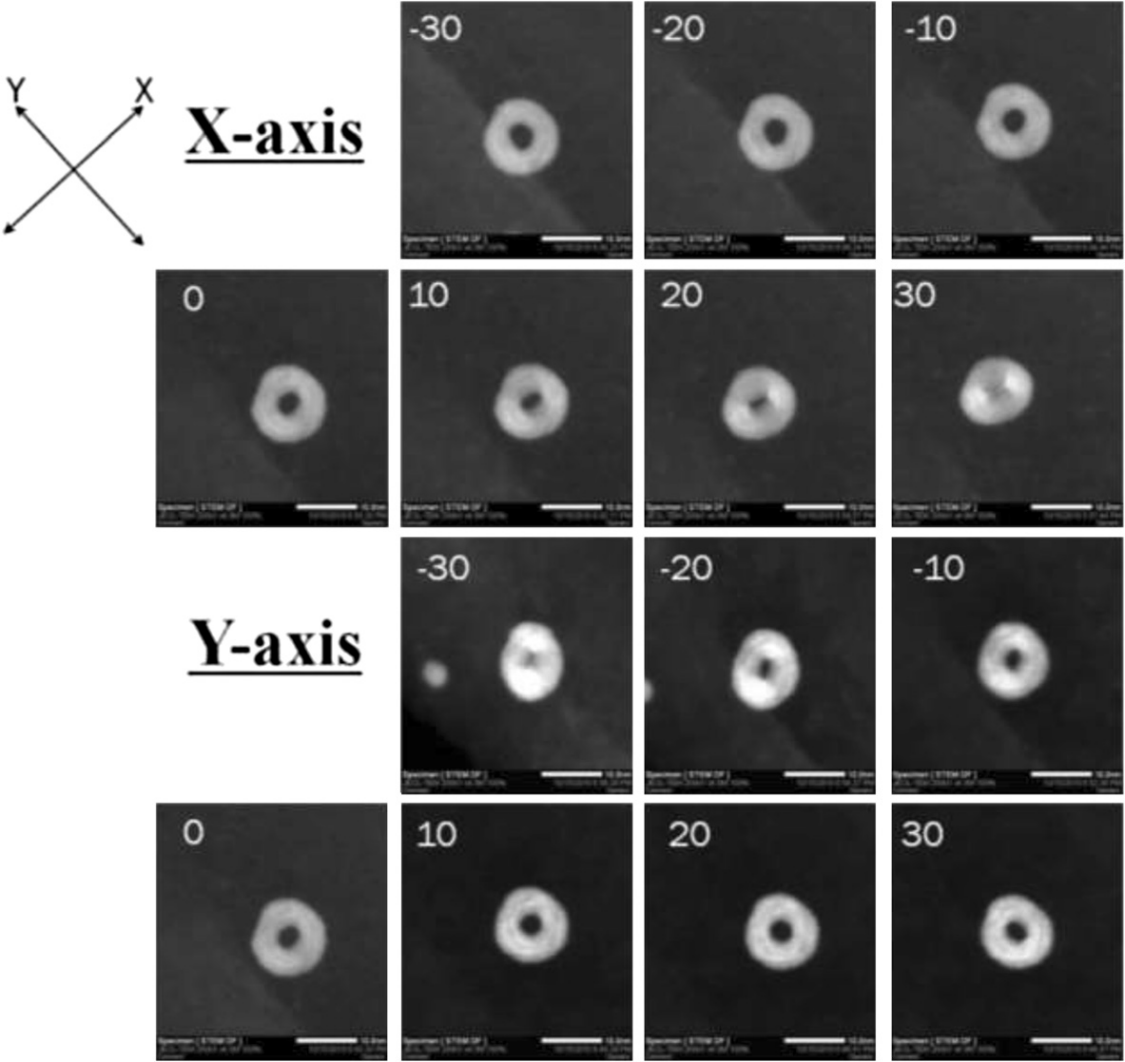
HAADF images of Au-Ag hollow particles with different rotation angles under STEM mode

**Fig. 7:**
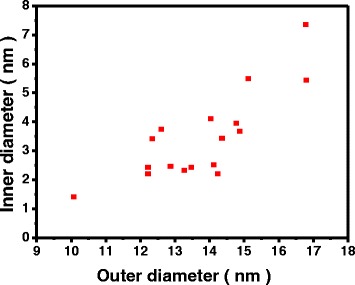
Outer diameters and inner diameters of the Au-Ag nanorings

The literature claims that the 0-D Au-Ag nanostructure formed through galvanic replacement between Ag atom and Au ions are mostly core-shell NPs or hollow spheres [6, 19, 22–30]. In order to clarify this issue, the 0-D hollow structures synthesized in this study were examined by HAADF-STEM images from different angles (Fig. 6). Observations from continuous rotation along *X* and *Y* axes ranging from −30° to 30°, verify that the hollow structures were rings rather than hollow spheres or core-shell particles. It can be speculated that previous studies might have misjudged the morphologies. For instance, the rings in the photos 30 rotated along the *X*-axis and −30° along the *Y*-axis resemble hollow or core-shell particles. In addition, a quantitative data estimating the inner and outer diameters of the alloy nanorings were obtained through TEM image analysis. Figure 7 indicates that the outer diameter of the nanorings ranged from 12~15 nm, while the inner diameter was between 2~4.5 nm.

**Fig. 8:**
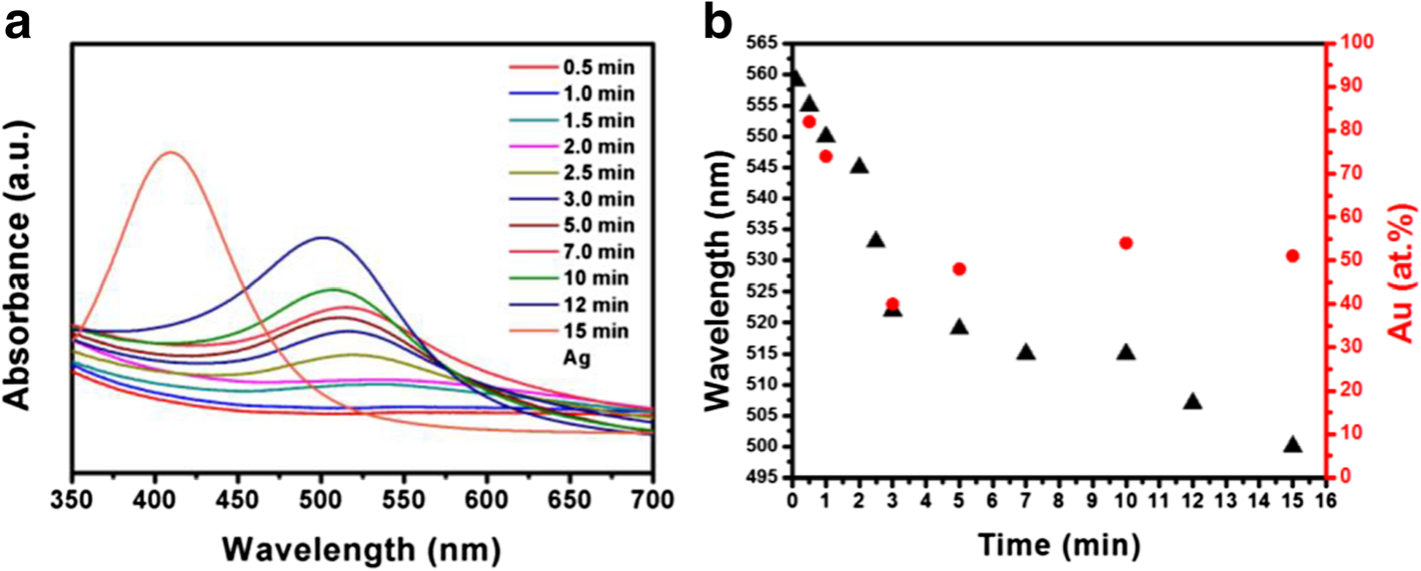
(**a**) UV-vis spectra and (**b**) wavelength of absorption peaks and Au content as a function of galvanic reaction time in the case of AuAg nanorings

By means of terminating the alloying process, the UV-vis absorption peaks of the 0-D nanostructure subjected to different periods of galvanic replacement reaction are illustrated in Fig. 8a. Figure 8b shows the wavelength of those absorption bands of the 0-D nanostructures and the corresponding Au content measured by EDS. It is surprising that at the first 10 s, the absorption peak located at 559 nm reveals the signal was from Au-rich nanostructures instead of Ag. The chemical composition data at 30 s showing ~82 at. % Au verifies this observation. With an increase in reaction time up to 3 min, the absorption red shifted rapidly to 523 nm, and the Au content decreased to about 40 at. % as well. After that, the absorption band continued to move toward the long wavelength side, but slowly. The Au content rose to 50 at. % and then remained constant.

In the present study, the redox reaction along with those for the anodic dissolution and cathodic reduction are given as follows [37]:

1 $$ 3\mathrm{A}\mathrm{g}\left(\mathrm{s}\right) + {{\mathrm{AuCl}}_4}^{-}\left(\mathrm{a}\mathrm{q}\right)\ \to\ \mathrm{A}\mathrm{u}\left(\mathrm{s}\right) + 3\mathrm{A}\mathrm{g}+\left(\mathrm{a}\mathrm{q}\right) + 4{\mathrm{Cl}}^{-}\left(\mathrm{a}\mathrm{q}\right) $$

Dissolution of Ag:

2 $$ 3\mathrm{A}\mathrm{g}\ \to\ 3{\mathrm{Ag}}^{+} + 3{\mathrm{e}}^{-}\kern2.25em {\mathrm{E}}_0 = -0.80\ \mathrm{v}\mathrm{v}\mathrm{s}\ \mathrm{SHE} $$

Reduction of Au ions:

3 $$ {\mathrm{AuCl}}^{-4} + 3{\mathrm{e}}^{-}\to\ \mathrm{A}\mathrm{u} + 4\ {\mathrm{Cl}}^{-}\kern0.5em {\mathrm{E}}_0 = 0.99\ \mathrm{v}\ \mathrm{v}\mathrm{s}\ \mathrm{SHE} $$

SHE: standard hydrogen electrode

**Fig. 9:**
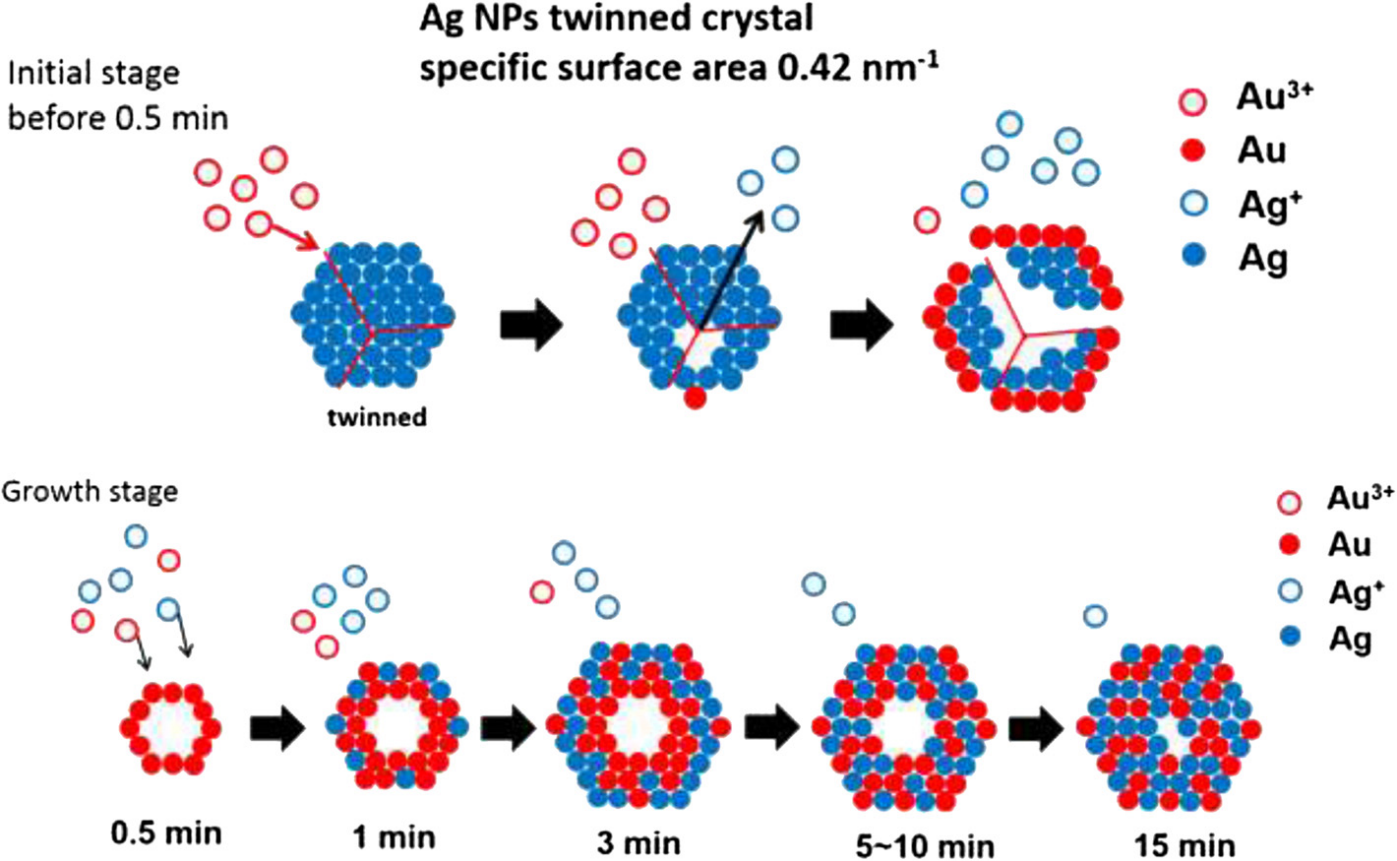
Mechanism for the formation of Au-Ag nanorings

The positive potential of the redox reaction gives rise to a spontaneous replacement of Ag by Au. However, due to the atomic valence, one Au atom replaces three Ag atoms [38], thus in the case of 0-D nanostructure, the Ag templates were consumed three times faster than the reduction and subsequent deposition of Au from the HAuCl_4_ solution. Therefore, as sketched in the upper diagrams in Fig. 9, Au ions obtained electrons from Ag atoms and thus rapidly dissociation of Ag NPs probably through the planar defects, i.e., twin boundaries in this case. The complete consumption of Ag along with the precipitation of Au atoms formed Au ring-like nanostructures. The subsequent co-precipitation of Au and Ag with the assistance of residual reducing agents, NaBH_4_ and Na_3_C_6_H_5_O_7_, as well as the interdiffusion of Au and Ag, gave rise to the formation and growth of alloy rings with an approximate Au/Ag ratio of 1. It has been demonstrated experimentally and by theoretical calculations that the surface plasmon resonance (SPR) of hollow Au NPs red-shifts significantly relative to the SPR of solid Au NPs of the same size [39]. In this study, the observation of absorption maximum for 0-D nanostructures at wavelengths value (506 nm) being red-shifted compared to that of Ag@AgAu core-shell solid structure [23] indicates that the 0-D nanostructures here may be hollow. The slow blue shift after 3 min of reaction might have resulted from the thickening and coarsening of the ring structure, gradually approaching solid AuAg NPs [25].

**Fig. 10:**
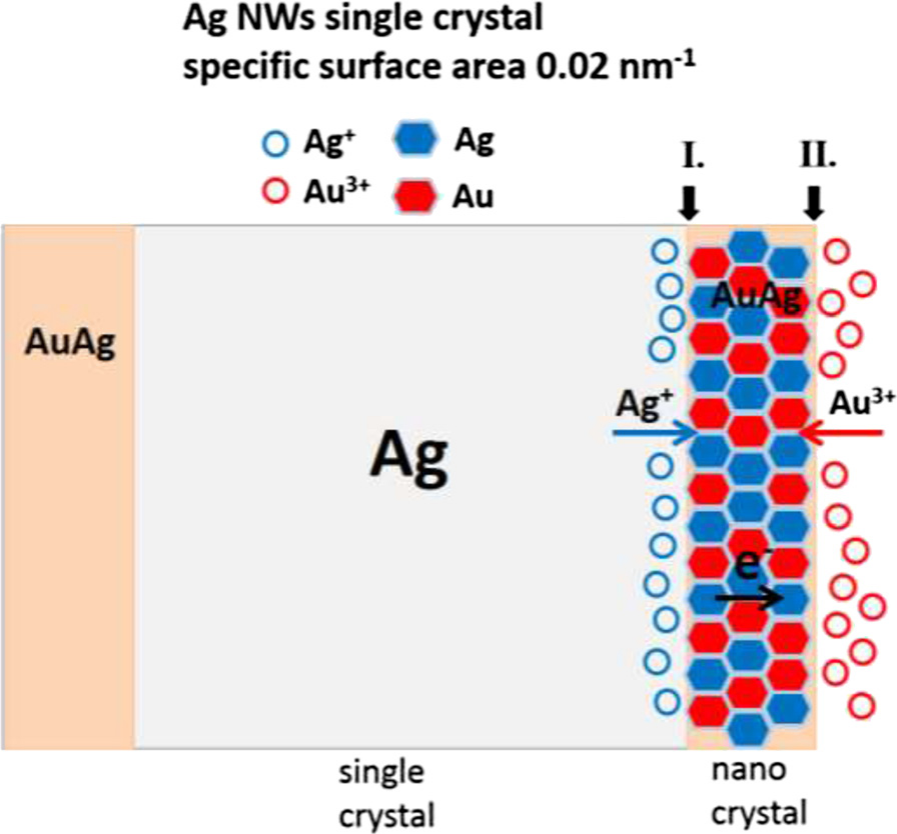
Mechanism for the formation of Ag@AuAg core-shell NWs

With respect to 1-D Au-Ag nanostructure, AuAg alloy nanocrystals were deposited on the surface of Ag wires as a compact layer of nanocrystals. We suggest that there may have been two interfaces, the inner surface of the nanocrystalline deposit between Ag and reduced Au (interface I in Fig. 10) and the other one, the outer surface between reduced Au and solution (interface II in Fig. 10). Dissociation of Ag (Eq. 2) can be considered to have occurred at interface I, while the reduction of Au took place at interface II (Eq. 3). There is no evidence so far showing the orientation relationship between single-crystalline Ag and nanocrystalline deposits. However, it can be inferred that boundaries in between nanocrystals provided large quantities of microchannels for the transportation of Ag ions, and thus Ag ions generated at interface I could transfer rapidly through the Au deposit. On the other hand, the electrons could travel through highly conductive deposits easily to support the reduction of Au ions at interface II. Meanwhile, it can be deduced that not only the galvanic reaction but also the interdiffusion of Au and Ag proceeded to turn Au deposits into AuAg alloy shell. The chemical composition shown in Table 2 led us to believe that the Au/Ag ratio of 1 is the stablest composition for interdiffusion products. This may be due to the isomorphous feature of this binary system (Au and Ag are mutual soluble at any composition) and the minimum free energy caused by the high entropy of mixing [40].

With the same concentrations of metallic ions in the precursor solutions, the selection of Ag templates caused quite different nanostructures, i.e., AuAg alloy hollow structure, more precisely, nanorings when Ag NPs were adopted as templates, and Ag@AuAg core-shell NWs in the case of Ag NW templates. Such differences can be attributed to two factors, specific surface area as well as the planar defects. Compared with Ag NWs (0.02 nm^−1^), a much greater specific surface area of Ag NPs (0.42 nm^−1^) may result in an accelerated kinetics for galvanic replacement. However, in comparison with the previous studies listed in Table 1, it can be deduced that the planar defects (twin boundaries) within the Ag templates are the key achieving a hollow structure instead of the size, shape, and the specific surface area. Subjected to galvanic replacement with sufficient Au ions, single-crystalline NPs/NWs turn into AuAg sold structures, while those with twins form a hollow alloy structure. Along twin boundaries, which are regarded as the fast diffusion path, the Ag templates decomposing from inside and being consumed at the very early stage are a prerequisite to the formation of the unique ring structure.

## Conclusions

The morphology and shape of Au-Ag nanostructures can be tuned through the selection of Ag templates in galvanic replacement process. Reacted with Au ions, Ag NPs turned into AuAg alloy rings with the Au/Ag ratio of 1. The characteristics of the alloy ring structure, as well as details of its formation were documented. In contrast, single-crystalline Ag NWs became Ag@AuAg core-shell wires instead of a hollow nanostructure. Combined with the relevant literature, it can be suggested that twin boundaries supporting fast diffusion path for the decomposition of Ag templates are the dominating factors in the formation of hollow/ring alloy nanostructures.
